# The Depth of Cure, Sorption and Solubility of Dual-Cured Bulk-Fill Restorative Materials

**DOI:** 10.3390/ma16206673

**Published:** 2023-10-13

**Authors:** Bashayer Alzahrani, Abdulrahman Alshabib, Wedad Awliya

**Affiliations:** Department of Restorative Dentistry, College of Dentistry, King Saud University P.O. Box 60169, Riyadh 11545, Saudi Arabia; wawliya@ksu.edu.sa

**Keywords:** dual cured, depth of cure, hardness, sorption, solubility

## Abstract

This study aimed to examine depth of cure (DoC), mass change, water sorption and solubility of dual-cured bulk-fill restorative materials (Surfil One and Activa) in comparison with a light-cured bulk-fill composite (Filtek One Bulk-Fill) and a resin-modified glass ionomer (Fuji II LC). Twenty specimens were prepared of each material using stainless steel molds designed with a slot (8 × 4 × 2 mm) and irradiated for either 20 or 40 s. The Vickers hardness (VHN) was measured at every 0.5 mm to assess the DoC after 24 h of storage at 37 °C. The depth of cure was reported as the depth corresponding to 80% of the maximum Vickers hardness. Disc-shaped specimens were prepared of each material (*n* = 5) to investigate mass change, sorption and solubility after 4 months of water storage. The data were analyzed using a two-way and one-way analysis of variance (ANOVA) followed by the Tukey post hoc test (*p* ≤ 0.05). Fuji II LC had the greatest DoC while Activa had the lowest. The two different irradiation times did not demonstrate a significant difference in DoC for all dual-cured materials (*p* > 0.05). Fuji II LC had the highest sorption while Filtek One showed the lowest. Surefil One and Fuji II LC had a negative solubility. This study concluded that dual-cured materials showed different depth of cure values despite having the same setting reaction. Both materials exhibited a high water sorption, which might jeopardize their dimensional stability and effect their clinical performance.

## 1. Introduction

Resin-based composites (RBCs) are widely preferred for direct restorations due to their adequate properties and excellent esthetic characteristics [1]. The recommended placement technique of a conventional composite is layering with increments of a 2 mm thickness. However, this technique is considered time-consuming and highly sensitive [2,3]. To address the need for a more efficient placement technique, light-cured bulk-fill composites were introduced, allowing for placement in a single increment of up to 4–5 mm. The deeper cure in bulk-fill composites is achieved mainly through alterations in translucency and photo-initiator systems [4]. Despite this advancement in the formulation of light-cured RBCs, insufficient polymerization is still a concern when restoring deep cavities. The limited depth of cure has been a significant challenge associated with light-cured RBCs, as noted in previous studies [5]. This limitation mainly arises from light attenuation due to absorption of monomers and photo-initiators within the material. Additionally, scattering and refraction at the filler/matrix interface contribute to more light attenuation. Previous data regarding the curing efficiency of bulk-fill composites in deep layers have been inconclusive, leading to uncertainty in this regard [4,5,6].

The mechanical properties of bulk-fill composites can vary considerably due to significant differences in their composition [4]. Previous data have indicated lower mechanical properties in most bulk-fill composites compared to conventional RBCs [7,8]. However, a systematic review assessing the clinical performance of bulk-fill composites concluded that there was no significant difference in failure rates compared to conventional RBCs [9].

Dual-cure resin composites for direct restorations were recently introduced. Dual-curing RBCs utilize two initiator systems, combining light-cure and chemical-cure components. The top layers of the material achieve initial hardening through the action of the light-cure initiator. However, for deeper layers that may not receive adequate light irradiation, polymerization is achieved through a chemical-cure reaction [10].

Through the combination of both light-cure and self-cure mechanisms, dual-cured restorative materials are presumed to overcome the challenge of limited depth of cure. Wang et al. assessed the degree of conversion and polymerization rate of dual- and light-cured RBCs at two different depths. They concluded that dual-cure composites generally exhibited a higher degree of conversion compared to light-cure bulk-fill composites [11]. Another study examined the degree of conversion and hardness of dual-cured bulk-fill composites. They reported that dual-cure bulk-fill RBCs demonstrated an increased degree of conversion and the ability to attain unlimited depth of cure [12]. Furthermore, some of these dual-cure materials offer additional appealing features, such as ion releasing and self-adhesiveness. However, incorporating these features may interfere with the setting reaction and compromise the polymerization efficiency [13,14].

The depth of cure (DoC) of resin composites is considered an indicator of the extent to which effective polymerization has been achieved within the material. There are several available methods for examining the depth of cure, including the scraping method as suggested with ISO standard 4049, optical microscopy and direct conversion measurement using mid- and near-infrared spectroscopy [15,16]. Concerns were raised regarding the suitability of the ISO 4049 test for accurately determining the DoC in bulk-fill RBCs. It was reported that the ISO 4049 test tends to overestimate the true DoC [17]. As an alternative, other techniques were commonly employed, such as hardness profile testing or top and bottom surface hardness assessment. The hardness profile technique involves measurement of microhardness cross-sectionally at different depth points until an 80% bottom-to-top ratio is achieved [18]. Previous studies indicated a linear correlation between the degree of conversion and microhardness values of RBCs [12,19].

The oral environment poses unique challenges to restorative materials due to its dynamic nature and exposure to various solvents, which can potentially be absorbed into the material. This interaction can cause the release of unreacted monomers from the material into the surrounding environment [20]. The fluid uptake and monomer release significantly affect physical and mechanical properties including strength, hardness, wear resistance, color and dimensional stability of restorative materials [21,22,23]. Therefore, the investigation of water sorption and solubility is crucial as these properties play a vital role in determining the long-term success of the restorative material.

Considering the increasing attention towards dual-cured materials for direct restorations, there is a need for further research in this area. Therefore, the objectives of this study were (i) to determine the depth of cure (DoC) of dual-cured materials, (ii) to evaluate the effect of irradiation time on DoC and (iii) to examine water mass change, sorption and solubility of these materials. The null hypotheses were as follows:

- There is no significant difference in the DoC between tested materials.
- There is no significant effect of the irradiation time on the Vickers hardness numbers and DoC of each individual material.
- There is no significant difference in mass change, water sorption or solubility among tested materials after 4 months of water storage.

## 2. Materials and Methods

Two dual-cured bulk-fill materials (Surefil One and Activa Bioactive) will be investigated in comparison to a light-cured bulk-fill (Filtek One Bulk Fill) and a resin-modified glass ionomer (Fuji II LC) (Table 1).

### 2.1. Study Design

For the first part of this study, the independent variables were the material type and the irradiation time. The dependent variables were (i) the maximum Vickers microhardness, (ii) 80% of the maximum Vickers microhardness and (iii) the depth at 80% of the maximum Vickers hardness.

For the second part, the independent variable was the material type, while the dependent variables are the mass change, water sorption and solubility.

### 2.2. Depth of Cure

**Figure 1:**
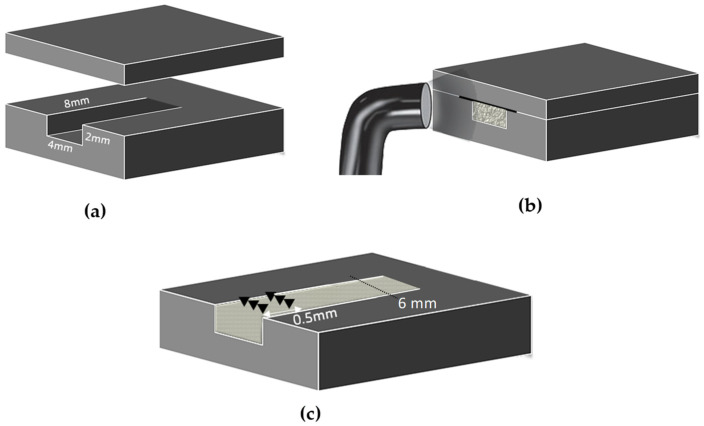
The stainless steel mold for the depth of cure specimens’ preparation. (**a**) The mold’s dimensions and the cover plate; (**b**) the tested material was placed and secured with the cover plate and Mylar strip in between (black line); and then a light-cured (**c**) illustration of the indentations (black triangles) for the Vickers hardness test.

The G*Power calculator was used for sample size estimation. A total of eighty specimens was determined to be sufficient at a significance level of 0.05, with an effect size of 0.5 and a power of 0.85. Using stainless steel molds, a total of 80 specimens (20 from each material) were prepared for the measurement of surface hardness profiles. The molds were specifically designed with a slot that had dimensions of 8 × 4 × 2 mm, along with a flat top cover as illustrated in Figure 1. Materials were dispensed into the mold until it was slightly overfilled. Then, a Mylar strip and the cover plate were positioned over the mold, and excess material was removed. The molds were irradiated from one end for either 20 s (according to the manufacturer recommendation) or 40 s. For light curing, a high-power (1200 mW/cm^2^) LED light-curing unit (Bluephase-G2, Ivoclar Vivadent, Schaan, Liechtenstein, Switzerland) was used. Prior to light curing each specimen, a calibrated radiometer (MARC™ Resin Calibrator, Blue-light Analytics Inc., Halifax, NS, Canada) was used to check the irradiance. At 37 °C, all specimens were stored in a dry incubator for 24 h before measurement. A micro-hardness instrument (FM-700, Kawasaki, Kanagawa, Japan) was used to create indentations on the specimen surface, starting from the top surface and subsequently at intervals of 0.5 mm. A 300 g constant load was applied for a duration of 15 s. The depth of cure was determined based on the following obtained values: the maximum Vickers microhardness, 80% of the maximum Vickers microhardness and the depth corresponding to 80% of the maximum Vickers hardness.

### 2.3. Water Sorption and Solubility

Following a modified version of ISO 4049, five specimens were made of each material using a brass mold of a 15 mm diameter and a 2 mm thickness [24]. The mold was positioned on top of a glass slide with a Mylar strip (Mylar Uni-strip, Caulk/Dentsply, Milford, DE, USA). The materials were dispensed into the mold, slightly overfilled and then covered with another Mylar strip and a 1-mm-thick glass slide. Slight pressure was applied to remove any excess material. A high-power (1200 mW/cm^2^) LED light-curing unit (Bluephase-G2, Ivoclar Vivadent, Schaan, Liechtenstein, Switzerland) was used to cure each specimen for 20 s using 5 overlapping sections from both top and bottom surfaces. After removing the specimens from the mold, any excess flash was eliminated using a silicon carbide paper of 1000 grit.

All specimens were placed in individual glass containers filled with fresh silica gel and then stored in a desiccator set at a temperature of 37 °C. The mass of each specimen was measured using a precise electronic microbalance (BM-252, AND company, Tokyo, Japan) with an accuracy of 0.01 mg. The measurements were taken until the change in mass over a 24 h period was less than 0.1 mg. Once a constant mass was reached ($m_{1}$), the diameter and thickness of each specimen were measured using a digital caliper. These measurements were used to calculate the volume (v) of each specimen according to this equation:

(1) $$v=\pi r^{2}h$$

where *π* = 3.14, (r) is the cross-section radius and (h) is the thickness of the specimen.

The specimens were then individually placed in a glass container with 10 mL of distilled water at 37 ± 1 °C. The specimens were weighed at different storage intervals 1 day, weekly for the first month and then every 2 weeks for 3 months (total of 4 months), and the obtained mass was denoted as ($m_{2}$). Prior to each measurement, the specimens were removed from the container, dried using filter paper, weighed and then returned to the storage media. The storage media was replaced every 7 days to ensure consistent pH levels. After the completion of the storage period, the specimens were subjected to a desorption cycle using storage in fresh silica gel to attain a constant mass ($m_{3}$). The percentage mass change during storage was calculated using the following formula:

(2) $$M\%={((m}_{2}-m_{1})/m_{1})\times 100$$

$m_{1}$ is the mass before water storage; $m_{2}$ is the mass after water storage for 4 months.

The following formulas were used to determine sorption (*SP*) and solubility (*SL*) (μg/mm^3^):

(3) $$M_{SP}={(m}_{2}-m_{3})/v$$

(4) $$M_{SL}={(m}_{1}-m_{3})/v$$

$m_{1}$ is the mass before water storage; $m_{2}$ is the mass after water storage for 4 months; $m_{3}$ is the mass after the desorption cycle; and v is the specimen’s volume.

### 2.4. Statistical Analysis

Data were analyzed using SPSS 24.0 statistical software (IBM SPSS Statistics, SPSS Inc., Chicago, IL, USA) with a significance level set at *p* ≤ 0.05. The mean values and standard deviations were calculated for the Vickers hardness, mass change, water sorption and solubility. Two-way ANOVA was used to assess the effect of depth and irradiation time on the values of VHN among study materials. One-way ANOVA, followed by Tukey post hoc, were performed to compare the mean values of Max. VHN, 80% Max. VHN and the depth of cure among the materials. One-way ANOVA was also performed to analyze the mass change, water sorption and solubility, followed by Tukey post hoc tests.

## 3. Results

### 3.1. Depth of Cure

**Figure 2:**
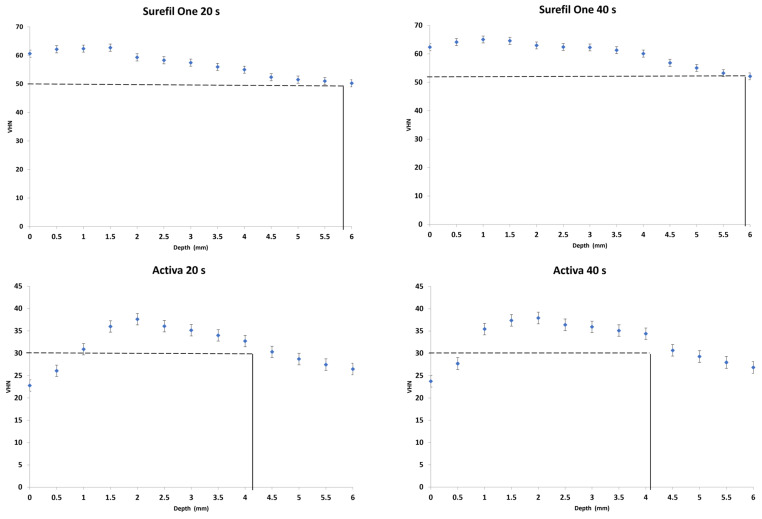

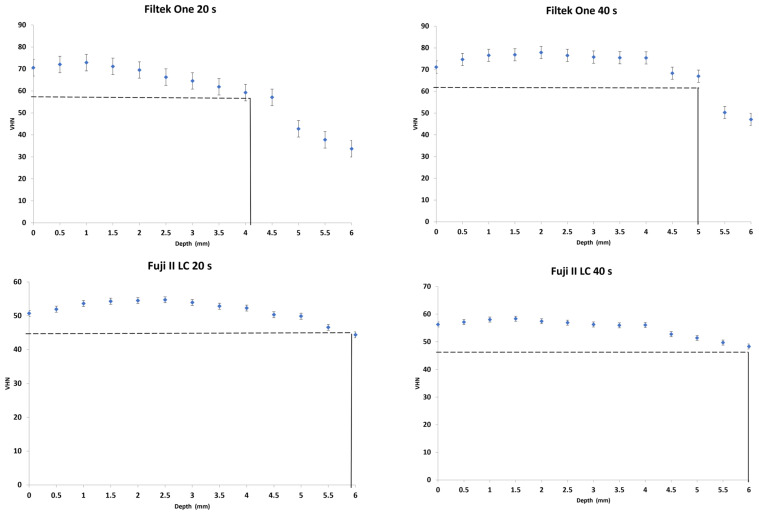
VHN at 80% of Max. VHN (dashed line) and depth at 80% of Max. VHN (solid line) of the tested material.

The Max. VHN, VHN at 80% of Max. VHN and depth of cure that corresponded to 80% of max. for each material at the different curing times are reported in Table 2. The mean VHN and standard deviation obtained at different depth points for each material are reported in Figure 2. Two-way ANOVA reported a significant difference in Max. VHN and VHN at 80% of Max. VHN of the tested materials among the two irradiation times (*p* = 0.000). 

One-way ANOVA showed statistically significant differences between materials for Max. VHN and VHN at 80% of Max. VHN (*p* = 0.000). For both irradiation times, the highest Max. VHN (78.1 ± 0.7) was observed in Filtek One, whereas Activa had the lowest (37.8 ± 0.7). The individual comparison of Max. VHN among all groups ranked the products: Filtek One > Surefil One > Fuji II LC > Activa. For DoC at the 20 s irradiation time, Fuji II LC had the greatest value (5.8 ± 0.4) with no significant difference with Surefil One (*p* = 0.8). Filtek One showed the lowest DoC value (4.1 ± 0.2), and there was no significant difference with Activa (4.2 ± 0.2) (*p* = 0.9).

The comparison of mean values of Max. VHN and 80% Max. VHN between the two irradiation time points demonstrated a statistically significant difference for all the tested materials except for Activa (*p* = 0.085). The mean values of Max. VHN and 80% Max. VHN were significantly higher at the 40 s irradiation time than the mean values at 20 s (*p* = 0.000). Comparisons of the DoC for each individual material showed that the two different irradiation times did not demonstrate a significant difference for Surefil One (*p* = 0.47), Activa (*p* = 0.66) and Fuji II LC (*p* = 0.17). However, light irradiation for 40 s showed a significantly greater DoC for Filtek One (*p* = 0.000).

### 3.2. Sorption and Solubility

**Figure 3:**
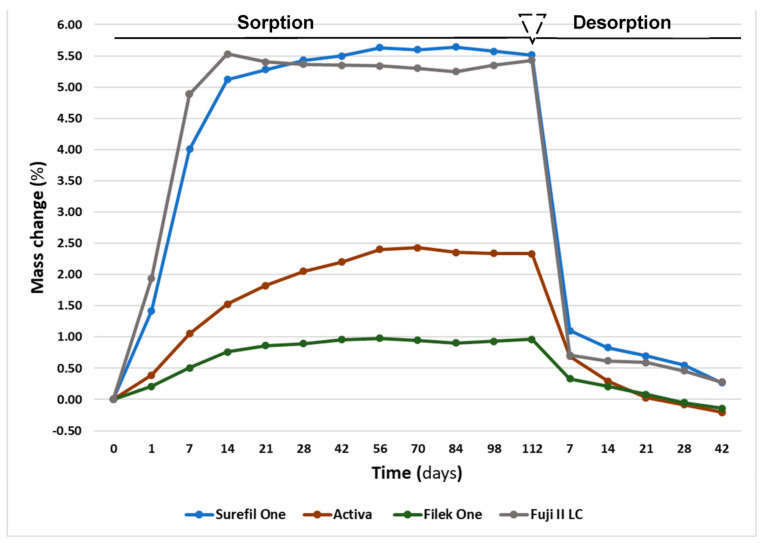
Mass change percentage with water sorption and desorption cycles.

The mean values for mass change, water sorption and solubility are demonstrated in Table 3. During the water sorption/desorption cycle, all the materials displayed a percentage mass change as demonstrated in Figure 3. The water uptake resulted in varying degrees of mass increase for all materials. Filtek One and Activa presented a higher initial mass ($m_{1}$) compared to their mass after desorption ($m_{3}$). Conversely, a higher final mass than the initial mass was demonstrated with Surefil One and Fuji II LC, with no significant difference between them (*p* = 0.73).

After 4 months, a statistically significant difference (*p* = 0.000) was observed when comparing the mean values of water sorption among the materials. The highest sorption was observed in Fuji II LC (108.7 µg/mm^3^ ± 1.5), followed by Surefil One (101.9 µg/mm^3^ ± 1.5), then Activa (44.2 µg/mm^3^ ± 1.3). Filtek One exhibited the lowest sorption (21.6 µg/mm^3^ ± 0.8).

The solubility values for the tested materials ranged from −5.8 to 2.8 µg/mm, as illustrated in Table 3. The most soluble material was Activa (5.4 µg/mm^3^ ± 0.4) followed by Filtek One (2.8 µg/mm^3^ ± 0.9). Negative solubility values were shown for Surefil One and Fuji II LC, with no significant difference between them (*p* = 0.98). 

## 4. Discussion

### 4.1. Depth of Cure

Several dual-cured materials are currently available to be used as direct restorations enabling bulk-fill placement without the need for capping with a conventional composite. Surefil One and Activa are novel products that claim to possess a dual-cure setting mechanism, improving the depth of cure. They are also promoted for their adequate mechanical properties and stability in the oral environment [25,26,27]. 

In this study, the depth of cure (DoC) of Surefil One and Activa was examined by measuring their hardness profiles at two clinically relevant irradiation times. A light-cured bulk-fill composite and a resin-modified glass ionomer were also investigated for comparisons. The results showed statistically significant differences in Max. VHN and DoC among the materials tested. Thus, the first null hypothesis was rejected. Additionally, the two different light irradiation times resulted in significantly different VHN values and DoC for some of the tested material. Hence, the second null hypothesis was partially rejected. 

The DoC of light-cured materials depends on the ability of visible light to penetrate through the material and provide the necessary energy for optimal polymerization. As a result, three key parameters need to be considered: adequate light output, sufficient exposure time and the proper wavelength range. Additional factors that can affect the DoC include the filler particle characteristics, the translucency of the material and the distance from the light-curing source. These factors collectively play a critical role in determining the extent of polymerization achieved within the material [28,29]. 

The exposure time is a crucial factor that affects the polymerization of RBCs’ restorations [28]. Some clinicians may elicit to extend the recommended exposure time in order to offset the reduction in light radiant power caused by unfavorable clinical conditions. Yap et al. evaluated the DoC of packable and flowable bulk-fill RBCs after irradiation for 20 s; the results showed that none of the tested materials maintained an 80% bottom-to-top hardness ratio at a depth of 4 mm [5]. AlQahtani et al. reported that a 4 mm DoC of the tested bulk-fill material was only achieved when curing was used for 40 s [30]. Furthermore, Zorzin et al. reported that extending the curing time beyond the manufacturer’s recommendations had a positive impact on the degree of conversion and the microhardness of the tested bulk-fill RBCs [31]. These findings suggest that increasing the light-curing time can enhance polymerization and mitigate the potential effects of insufficient light output.

In this study, Filtek One demonstrated the highest Max. VHN values, followed by Surefil One, which was expected because of their high filler content. Previous studies reported a linear correlation between VHN and filler loading [32,33]. Despite high VHN values, Filtek One presented the lowest DoC when cured for 20 s. This might indicate inadequate polymerization in deep layers and raise doubts regarding the manufacturer’s assertion of achieving a DoC of up to 5 mm. However, when irradiated for 40 s, Filtek One exhibited significantly higher VHN values and achieved a DoC up to 5 mm. Similar findings were reported in previous studies [12,34].

Fuji II LC demonstrated a notably high DoC of 5.7 and 6 mm after light exposure times of 20 and 40 s, respectively. These values were significantly higher than the manufacturer’s recommended DoC [35]. Previous studies conducted on Fuji II LC also reported similar findings [36,37,38]. Surefil One and Fuji II LC presented no significant difference in DoC. This is obviously attributed to the fact that these materials own a self-curing mechanism and do not rely solely on light for polymerization of the deep layers. 

Activa had the lowest VHN values, which can be explained with its low filler load (56%) and the presence of bioactive glass fillers (BAG), which have been shown to reduce surface hardness in previous studies [39,40]. Activa also had the lowest DoC (4 mm) in comparison to the other dual-cure materials in this study. This result meets the manufacturer-reported DoC and is in agreement with previous studies [36,41]. Despite the manufacturer’s report that it is a dual-cure material, Activa may have a less effective chemical-cure mechanism compared to other dual-cured materials. Hughes et al. also reported that the self-curing characteristic of Activa was shown to be limited [42]. This limitation in DoC might also be attributed to the BAG filler load as several studies observed a decrease in the degree of conversion with higher concentrations of BAG fillers. This reduction could be attributed to factors such as light scattering or interference with the polymerization process with inactivation of the free radicals [43]. 

### 4.2. Water Sorption and Solubility

In this study, the water sorption and solubility of the tested materials were evaluated over a period of 4 months. The results reported significant differences among the tested materials. Consequently, the null hypothesis stating that there is no difference in mass change, water sorption and solubility between the tested materials was rejected. According to ISO standard 4049, the sorption limit is 40 µg/mm after 7 days of water storage. However, considering the extended storage period in this study, all the materials except Filtek One exceeded the sorption limit. Nevertheless, all the materials showed acceptable solubility behavior below 7.5 µg/mm^3^, which is the maximum water solubility stated with ISO 4049 [15].

The hydrophilicity and the extent of cross-linking in the network structure are the main factors that impact the sorption and solubility of restorative materials. The amount of solvent uptake during the exposure time is also influenced by factors such as the presence of porosity within the material and the nature of the filler and filler/matrix interface [20]. Water sorption is a diffusion-controlled process that is regulated through two different mechanisms. The free volume is considered as the first approach where the water molecules are collected at filler/matrix interfaces and in intermolecular spaces. The second approach is the interaction of water molecules and hydrophilic groups to form hydrogen bonds [44].

In the oral environment, solvents and chemical agents can be absorbed by the resin matrix accompanied by leaching out of unreacted monomers [45]. Over time, water penetration could result in hygroscopic expansion, stress and microcracks. This damage to the internal structure accelerates water uptake and promotes further degradation. Moreover, water has a plasticization effect on the resin matrix network that negatively impacts mechanical and physical properties [46]. Therefore, water sorption and solubility behavior of RBC materials is of significant interest as it affects the material’s properties, hence, longevity and failure rates.

In this study, it was expected that the RMGI material (Fuji II LC) would exhibit the highest water uptake. This material undergoes a dual-setting reaction involving mainly the acid–base reaction of conventional glass-ionomer cement and the free radical polymerization process of methacrylate monomers. The hydrophilic nature of the polymerized structure added in Fuji II LC, which includes HEMA, results in a matrix structure that contains numerous hydrophilic functional groups. The filler particles become involved in the formation of a hydrogel structure that can absorb an extensive amount of water. Therefore, water uptake is not confined solely to the matrix, but impacts the entire hydrogel structure, resulting in an accelerated water uptake [47].

Surefil One presented a comparable sorption value to Fuji II LC, which could be due to similar aspects of their composition. Surefil One is composed of a high load of fillers, mainly aluminum-fluoro-silicate glass, and the matrix consists of bifunctional acrylate and modified polyacids functionalized with polymerizable groups. It was reported to offer a self-adhesive property [48]. A study investigating this material revealed a high phosphate content that was linked to the bifunctional acrylate, possibly a phosphate-based functional monomer incorporated to promote adhesion [49]. Earlier studies reported that self-adhesive composites with an acidic functional group demonstrated high solvent sorption and substantial expansion [24,50]. Moreover, a certain proportion of water presents in the composition to act as a solvent for a polyacid and resins and to aid in the self-adhesiveness [49]. All these factors along with the acid–base reaction and hydrogel structure formation may have contributed to the high sorption value. 

In this study, Activa showed a sorption value that exceeded the ISO limit, which can be explained with the filler/matrix system. The matrix is composed of a blend of diurethane and methacrylates with modified polyacrylic acid and phosphate acid groups as reported by the manufacturer [26]. Hydrophilic components such as polyacrylic acid and phosphate groups showed as increasing water sorption [51,52]. Moreover, the matrix structure in materials with BAG fillers must allow water to reach the particles and permit leaching out of ions [53]. In a study evaluating the effect of BAG on sorption and solubility of RBCs, the results showed that composites with 40% BAG fillers had six times higher water sorption compared to composites containing no BAG [54]. The solubility value for Activa was within the ISO limit despite a high sorption value. This might be explained with the degree of cross-linking. The trivalent ions released such as aluminum contribute in the cross-linking through an ionic bond between dimethacrylate phosphate and acrylic resin in the matrix [25]. 

The lower mass change, sorption and solubility values observed in Filtek One can be attributed to its high filler load and the hydrophobic nature of its highly cross-linked resin matrix in comparison to the other materials. In a highly cross-linked network, less free volume is available for penetration of the solvent, which increases the material resistance to the softening effect of solvents [55]. 

Surefil One and Fuji II LC had negative solubility values. Previous studies reported negative solubility in certain materials. This was attributed to the establishment of hydrogen bonds between the polymer chains and the water molecules absorbed in [56]. These hydrogen bonds are resistant to removal through dehydration and hinder additional water penetration. A previous study showed that with the progression of the acid–base reaction, the percentage of tightly bound water increased. This was elucidated by two possible mechanisms: the creation of highly hydrated ions or the development of a stable hydration sheath surrounding the ionized polyacrylic polymer [57]. Another possible explanation for the negative solubility is that these materials exhibited substantial water sorption, which potentially masked their actual solubility. 

According to the manufacturer, Surefil One contains hydrolytically stable amide cross-linkers that polymerize with all components of the formulation. In a study investigating the structure and chemical composition of Surefil One, a filler element analysis showed a percentage of nitrogen, which suggested that this material contains an acrylamide monomer. This might contribute to enhanced degradation resistance and thus a low solubility value [49].

Like other in vitro studies, this investigation has certain limitations. One limitation is that all the tested materials utilized the same shades and were cured using the same light-curing unit. Further testing options could include evaluating the depth of cure using different shades, employing different types of light-curing units and assessing the materials at various time points after irradiation. In order to expand the scope of the investigation, additional recently developed dual-cured bulk-fill materials from different brands could be incorporated and subjected to the same testing parameters. Further research is also recommended to evaluate the sorption and solubility of the materials in different solvents. The solubility and sorption test ISO-4049 is primarily designed for resin composite materials. Despite that all the tested materials include a resin component, care must be taken when interpreting the test findings for materials that include an acid–base reaction and have water in their structure. 

## 5. Conclusions

Within the limitation of this study, it can be concluded that

1. Surefil One achieved a 6 mm depth of cure whereas Activa showed 4 mm.
2. Increasing irradiation time produced significantly higher VHN values yet showed no effect on the depth of cure of dual-cured bulk-fill materials.
3. Both dual-cured bulk-fill materials exhibited higher mass change and water sorption values compared to the light-cured material.

## Figures and Tables

**Table 1:** Composition of the tested materials.

Material	Manufacturer	Lot Number	Resin Matrix	Filler Type	Filler Load wt%
Surefil One (Dual-cured)	Dentsply Sirona; Konstanz, Germany	2129000667	Modified polyacids (MOPOS), bifunctional acrylate, acrylic acid, BADEP, camphorquinone, self-cure initiator, stabilizer	Aluminum-phoshor-strontium-sodium- fluoro-silicate glass, highly dispersed silicon dioxide, ytterbium fluoride	77%
Activa Bioactive-Restorative (Dual-cured)	Pulpdent, Watertown, MA, USA	215573	Diurethane modified with hydrogenated polybutadiene, methacrylate monomers, modified polyacrylic acid	Bioactive glass, silica, sodium fluoride	56%
Filtek One Bulk-fill (Light-cured)	3M ESPE, St. Paul, MN, USA	NE19225	AUDMA, UDMA, diurethane-DMA, and DDDMA, AFM, camphorquinone	20 nm silica, 4–11 nm zirconia, cluster Zr-silica, 100 nm ytterbium trifluoride	76.5%
Fuji II LC (Dual-cured)	GC, Tokyo, Japan	225481A	HEMA, polyacrylic acid, UDMA, dimethacrylate	Alumino-fluoro-silicate glass	58%

RMGI: resin-modified glass ionomer, BADEP: N, N′-diethyl-1,3-bisacrylamido-propan, AUDMA: aromatic urethane dimethacrylate, UDMA: urethane dimethacrylate, DDDMA (1, 12-dodecanediol dimethacrylate), AFM: addition-fragmentation monomer, HEMA: 2-hydroxyethyl methacrylate.

**Table 2:** Mean (standard deviation) of Max. VHN, VHN at 80% of Max. VHN and depth at 80% of Max. VHN for the tested materials at 20 and 40 s curing time.

Material	Irradiation Time					
	20 s			40 s		
	Max. VHN Mean (SD)	VHN at 80% of Max. VHN Mean (SD)	Depth (mm) at 80% of Max. VHN Mean (SD)	Max. VHN Mean (SD)	VHN at 80% of Max. VHN Mean (SD)	Depth (mm) at 80% of Max. VHN Mean (SD)
**Surefil One**	63.3 (0.5) ^a,A^	50.6 (0.4) ^a,A^	5.8 (0.4) ^a,A^	65.1 (0.6) ^a,B^	52.1 (0.5) ^a,B^	5.9 (0.4) ^a,B^
**Activa**	37.8 (0.7) ^b,A^	30.3 (0.6) ^b,A^	4.2 (0.2) ^b,A^	38.4 (0.8) ^b,A^	30.7 (0.6) ^b,A^	4.2 (0.3) ^b,A^
**Filtek One**	72.8 (0.9) ^c,A^	58.2 (0.7) ^c,A^	4.1 (0.2) ^b,A^	78.1 (0.7) ^c,B^	62.5 (0.5) ^c,B^	5.0 (0.0) ^c,B^
**Fuji II LC**	55.4 (0.4) ^d,A^	44.3 (0.3) ^d,A^	5.9 (0.3) ^a,A^	58.6 (0.5) ^d,B^	46.9 (0.4) ^d,B^	6.0 (0.0) ^a,A^

For each column, the same superscript lowercase letter denotes that there is no significant difference (*p* > 0.05) in mean values between different materials. For each row, the same superscript uppercase letter denotes that there is no significant difference (*p* > 0.05) in mean values within each material among the two irradiation times.

**Table 3:** Percentage mass change, Water sorption (Wso) and solubility (Sol) of tested materials after 4 months of storage in distilled water.

Materials	Mass Change %	Wso (µg/mm^3^)	Sol (µg/mm^3^)
Surefil One	5.5 (0.3) ^a^	101.9 (1.5) ^a^	−5.1 (1.0) ^a^
Activa	2.3 (0.1) ^b^	44.2 (1.3) ^b^	5.4 (0.4) ^b^
Filtek One	1.0 (0.0) ^c^	21.6 (0.8) ^c^	2.8 (0.9) ^c^
Fuji II LC	5.4 (0.1) ^a^	108.7 (1.5) ^d^	−5.8 (1.6) ^a^

For each column, the same superscript letter indicates no significant difference (*p* > 0.05) in mean values between different materials.

## Data Availability

The article includes all data that support the findings of this study.
